# Are ROME Criteria for IBS Useful in Primary Care Practice?

**DOI:** 10.1002/ueg2.70289

**Published:** 2026-09-02

**Authors:** Niek J. de Wit, A. Pali S. Hungin

**Affiliations:** ^1^ Julius Center for Health Sciences and Primary Care University Medical Center Utrecht Utrecht the Netherlands; ^2^ Faculty of Medical Sciences Newcastle University Newcastle Upon Tyne UK

In this issue of UEG Journal Wils et al. present a secondary analysis of the DOMINO trial [1]. In the original RCT with 470 primary care patients newly diagnosed with IBS, the self‐managed FODMAP diet proved to be superior to spasmolytic medication [2]. The cross‐sectional analysis presented here focusses on the characteristics of the study population as a representation of the IBS patients in primary care. In addition, the authors assessed which factors predicted IBS severity.

The role of the ROME criteria was interesting. The authors found that ROME positive patients had higher symptoms scores, experienced more impact on quality of life and had higher symptom scores for anxiety and depression. In multivariate analysis ROME defined stool type, lower age and higher somatic symptom scores were associated with greater IBS symptom impact.

Authors conclude that the ROME criteria adequately identify IBS subtypes and discriminate against IBS disease severity. They suggest that in primary care practice the Rome criteria can be helpful to guide personalised treatment.

Although frequent in primary care, presentation of IBS is heterogeneous [3]. The key symptoms, abdominal pain and an altered stool pattern, present with different intensity, subjective impact, and accompanying symptoms. Also, although patients with IBS often have an increased psycho‐social burden, most are not clinically anxious or depressed, nor severely handicapped by their symptoms.

Primary care management of IBS is stepped, starting with a simple explanation and dietary advice for the majority, and medication, psychological and dietary interventions for the more severely affected [4]. Management steps are guided by the symptom impact as experienced by the patient. It is up to the general practitioner to assess these symptoms and their effect on quality of life, in the context of the patient's individual history and profile.

The ROME criteria have successfully helped to harmonize inclusion criteria for IBS patients in clinical research [5]. However, their relevance for clinical primary care practice is questioned by many. Most GPs do not use the ROME criteria, many are not familiar with them [6], and primary care guidelines for IBS, such as the NICE guidelines, do not contain specific advice on using them [4].

General practitioners generally base their clinical management on an overall impression of their patient, looking at somatic, psychological and social aspects. The ROME criteria may help to map the IBS status in detail, but management decisions will be based on the integrated impression of the symptoms, in the personal context of the patient.

In addition, the interpretation of symptoms by the GP may differ from the outcome of the ROME questionnaire. This is demonstrated by the results of the DOMINO study: one‐third of the patients did not meet the ROME criteria for IBS although they all had IBS according to the GP by the time of inclusion for the study. In more detail: although almost all patients had abdominal pain, in ROME negative patients the frequency of pain and the labelling of the stool type as interpreted by the GP did not meet the expert‐based ROME criteria. That underlines the difference between an expert‐based interpretation in clinical practice and an evidence‐based definition in symptom surveys.

One of the limitations of defining single specialty symptom clusters is their restricted ability to encompass the patient as a whole. Although the ROME Foundation has sought to refocus the concept of DGBI, relatively little attention is paid to the wide range of associated problems and co‐morbidities that patients commonly present with. DGBI rarely occur in isolation and are frequently accompanied by urological, gynaecological, musculoskeletal, or neurological symptoms. Despite this, specialist care is often delivered through the narrow perspective of an individual specialty. Such an approach risks compromising effective management from the outset. A legitimate question, therefore, is whether the use of ROME V criteria can meaningfully advance the concept of truly holistic patient care.

Ultimately, DGBI should be regarded as one component of a broader pattern of whole‐person dysfunction rather than an isolated disease entity. In many cases, the generalist clinician—most commonly the family doctor or general practitioner is best placed to assess the patient's overall clinical picture without being constrained by the finer points of DGBI classification. Specialist input remains indispensable for confirming diagnoses and directing management in case of more complex presentation of gastrointestinal symptoms. Nevertheless, a more pragmatic, collaborative, and integrated model of care would better reflect the realities of clinical practice.

Rather than directing other disciplines from within a gastroenterology specialist framework, future iterations of the ROME criteria could place greater emphasis on a more holistic approach, genuinely patient‐centred care and multidisciplinary collaboration.

## Conflicts of Interest

The authors declare no conflicts of interest.

## Data Availability

Data sharing not applicable to this article as no datasets were generated or analysed during the current study.
